# Validation of a portable nitric oxide analyzer for screening in primary ciliary dyskinesias

**DOI:** 10.1186/1471-2466-14-18

**Published:** 2014-02-10

**Authors:** Amanda Harris, Esther Bhullar, Kerry Gove, Rhiannon Joslin, Jennifer Pelling, Hazel J Evans, Woolf T Walker, Jane S Lucas

**Affiliations:** 1 Primary Ciliary Dyskinesia Centre, University Hospital Southampton NHS Foundation Trust, Southampton, UK; 2 NIHR Southampton Respiratory Biomedical Research Unit, University of Southampton and University Hospital Southampton NHS Foundation Trust, Southampton, UK; 3 Clinical and Experimental Sciences Academic Unit (Mail Point 803), University of Southampton Faculty of Medicine, University Hospital Southampton NHS Foundation Trust, Tremona Road, Southampton SO16 6YD, UK

**Keywords:** Nasal nitric oxide, Primary ciliary dyskinesia, Nitric oxide analyser

## Abstract

**Background:**

Nasal nitric oxide (nNO) levels are very low in primary ciliary dyskinesia (PCD) and it is used as a screening test.

**Methods:**

We assessed the reliability and usability of a hand-held analyser in comparison to a stationary nitric oxide (NO) analyser in 50 participants (15 healthy, 13 PCD, 22 other respiratory diseases; age 6–79 years). Nasal NO was measured using a stationary NO analyser during a breath-holding maneuver, and using a hand-held analyser during tidal breathing, sampling at 2 ml/sec or 5 ml/sec. The three methods were compared for their specificity and sensitivity as a screen for PCD, their success rate in different age groups, within subject repeatability and acceptability. Correlation between methods was assessed.

**Results:**

Valid nNO measurements were obtained in 94% of participants using the stationary analyser, 96% using the hand-held analyser at 5 ml/sec and 76% at 2 ml/sec. The hand-held device at 5 ml/sec had excellent sensitivity and specificity as a screening test for PCD during tidal breathing (cut-off of 30 nL/min,100% sensitivity, >95% specificity). The cut-off using the stationary analyser during breath-hold was 38 nL/min (100% sensitivity, 95% specificity). The stationary and hand-held analyser (5 ml/sec) showed reasonable within-subject repeatability(% coefficient of variation = 15).

**Conclusion:**

The hand-held NO analyser provides a promising screening tool for PCD.

## Background

Primary ciliary dyskinesia (PCD) is an autosomal recessive condition in which abnormal ciliary function leads to impaired mucociliary clearance and consequent recurrent upper and lower respiratory tract infection [1]. Approximately half the patients have *situs inversus* and male infertility is common [1]. Early diagnosis is important to ensure appropriate management and counselling; several studies suggest that early diagnosis and treatment improves long-term prognosis. To comply with European consensus guidelines diagnosis of PCD includes investigations requiring access to highly specialised equipment and diagnostic scientists [2-4]. Such facilities are not widely available, contributing to the inequality of diagnosed cases throughout Europe [5]. Nasal NO (nNO) is characteristically low in PCD [6,7], so much so that nNO is recommended as a pre-diagnostic screening test for the condition [2,3,8-10]. Until recently, the only commercially available analyzers for measuring nNO were non-portable desktop analyzers which are extremely expensive. A reasonably priced, practical method of measuring nNO might improve the disparity in diagnosis and reduce diagnostic costs by reliably identifying patients for referral to specialist centres. A number of hand-held devices have proved beneficial for measuring fractional exhaled nitric oxide in patients with asthma [11-14] in clinical and research settings. Commercially available hand-held analyzers have recently been adapted to measure nNO in breath-hold and tidal breathing modes [15,16]. A study of PCD and CF patients reported a hand-held device to be as effective as the stationary analyser for assessing nNO during silent and humming exhalation [17]. Furthermore, a recent Danish study has showed that tidal breathing nNO measured by a hand-held NO device discriminates between PCD, cystic fibrosis (CF) and healthy controls (HC) [18]. PCD is rare and no center has access to large numbers of patients. The two published studies using hand-held devices have respectively assessed nNO in only 14 PCD patients during silent and humming single breath exhalation [17] and 16 PCD patients during tidal breathing and during a velum closure maneuver [18]. Our data study therefore adds to the limited data, using a similar tidal breathing maneuver to that used in the Danish study. The aim of our single center cross-sectional study was to evaluate the ability of a commercially available analyzer (NIOX MINO®) to discriminate between PCD, other respiratory diseases and healthy controls in comparison to a ‘gold standard’ chemiluminescence analyser (NIOX® Flex).

## Methods

This study was approved by Southampton and South West Hampshire Research Ethics Committee A (06/Q1702/109 and 08/H0502/126). All subjects gave written informed consent.

### Participants

Fifty people consented to participate in this study, including 15 healthy volunteers, 13 with PCD and 22 with other respiratory disorders. Children and adults with PCD, cystic fibrosis (CF) (n = 6), and non-CF non-PCD chronic suppurative lung disease (CSLD) (n = 7) were recruited from specialist PCD and respiratory clinics. Those with asthma +/− hay fever (n = 9) were recruited from amongst staff/students and from respiratory clinics.

Healthy participants completed a short questionnaire to exclude disease that might affect nitric oxide levels. PCD was diagnosed at Southampton’s national PCD centre by analysing respiratory epithelia ciliary beat frequency and pattern using high-speed video microscopy in patients with a suggestive history [2,4,19]. Diagnosis was supported by assessment of ciliary ultrastructure by transmission electron microscopy (TEM). In some cases diagnosis was further clarified by analysis of re-differentiated cilia following culture of the airway epithelial cells at an air liquid interface. CF diagnosis was based on compatible history, an abnormal sweat test and/or CF genotyping. CSLD participants were recruited from patients with a chronic history of purulent sputum referred to the PCD diagnostic service who had the diagnosis of PCD and CF excluded. They had not necessarily had a HRCT; bronchiectasis had therefore not been radiologically excluded nor confirmed. Asthma diagnosis was self-reported amongst staff and students.

### Measurement of airway nitric oxide

Two different devices were used: NIOX MINO® and NIOX® Flex analysers. Measurement of nNO levels using NIOX® Flex (Aerocrine, Sweden) uses chemiluminescence with sampling at 5 ml/s [20], whilst the NIOX MINO® uses an electrochemical sensor and provides a result after sampling for 90 seconds at 2 ml/s or for 45 seconds at 5 ml/s. The analyser only provides a result if uninterrupted sampling has been maintained throughout this sampling time. NIOX MINO® can also sample during tidal breathing.

The NIOX® Flex analyser was maintained, calibrated and tested according to manufacturer’s guidelines. Measurements were made by health care professionals who had been trained following a standard operating procedure derived from the manufacturer’s guidelines.

In brief, ambient NO was noted before testing patients. All patients were symptomatically free of viral infection or respiratory exacerbation. Participants were requested to blow their nose before testing. A nasal probe sampled gas aspirated from the nostril at a rate of 5 ml/sec during a breath-holding maneuver. Patients held their breath for approximately 20 seconds until the real-time analyser recorded a plateau in nitric oxide concentrated from the aspirated gas. The measurement was recorded from a steady nNO concentration plateau of at least 4 seconds. Three measurements within 10% were obtained for each participant using the same nostril and the maximum nNO reading was recorded. nNO concentration in parts per billion (ppb) were converted to NO production (nL/min) using the equation NO production (nL/min) = nNO concentration (ppb) x sampling flow rate (litres/min).

The NIOX MINO® was used in nasal mode. A nasal probe sampled gas aspirated from the nostril at 5mls/sec and was then repeated at 2 ml/sec. Following ATS [20] and manufacturer’s guidelines we initially attempted these measurements during breath-holding manoeuvres To obtain a test result the participant needed to achieve uninterrupted sampling for 45 seconds whilst sampling at 5 ml/sec or 90 seconds at 2 ml/sec. Breath holding for this duration was not achievable even by healthy adult volunteers and it was decided to abandon this maneuver. Instead we followed the alternative method suggested by the manufacturers of sampling nasal gas at 2 ml/sec and 5 ml/sec during open mouth breathing. This non-velum closure technique has previously been confirmed as reproducible and valid using a stationary chemiluminescence analyser [21]. Three recordings at each rate were attempted, and the highest was taken.

### Usability and reliability of analysers

The multidisciplinary clinical PCD team who undertook these measurements (paediatrician, PCD nurse specialist, PCD respiratory physiotherapist and respiratory technicians) discussed the pros and cons of the analysers. Their consensus opinions were recorded.. The success rate for obtaining a measurement with each analyser was recorded.

### Statistical analyses

The highest of three measurements for each method was recorded. Nasal NO values were log_10_ transformed where appropriate because data was not normally distributed. Comparisons between disease groups were made using unpaired t-tests. Comparisons between analyzers were made using paired t-tests. Receiver Operating Characteristic (ROC) curves were used to determine cut of values, sensitivity and specificity. Pearson correlation coefficients were calculated to investigate correlation between nNO values obtained with the NIOX® Flex and NIOX MINO®^..^ Brand Altman plots were constructed with the difference of measurements from the two analyzers plotted against the mean of the two methods. The reproducibility of analyzers was reported as the intra-subject% Coefficient of Variability (%CV); this was calculated by calculating the standard deviation for the three measurements taken using each protocol, and dividing that by the triplicate mean, and multiplying by 100. Data were analyzed using statistical analysis software SPSS version 19.0.0 (IBM, USA).

## Results

Valid readings were obtained in 47 participants using the NIOX® Flex breath-hold protocol. Three participants (aged 5, 8 and 8 years) were unable to breath-hold for 20 seconds to obtain a technically acceptable measurement. Using the NIOX MINO® healthy adults were unable to manage the breath-hold protocol of 45 seconds for 5 ml/sec or 90 seconds for 2 ml/sec and we abandoned these protocols with no resulting data. However measurement during tidal mouth breathing using the NIOX MINO® successfully provided data for 48 participants at a sampling rate of 5 ml/sec and 38 participants at 2 ml/sec. The mean (range) ages of participants in each group were: healthy controls 31 years (8–65), PCD 23 years (5–71), CF 15 years (6–29), asthma 35 years (12–59) and CSLD 36 years (8–79).

### Nasal NO as a screening tool for PCD

Using the NIOX® Flex, patients with PCD had significantly lower levels of nNO than healthy controls (p < 0.001), CF (p = 0.003), asthma (p < 0.001) and CSLD (p = 0.005). Statistically lower nNO values were similarly obtained in patients with PCD when using the NIOX MINO® at 5 ml/sec (HC p < 0.001; CF p = 0.028; asthma p < 0.001; CSLD p = 0.002). Using the NIOX MINO® sampling at 2 ml/ sec, patients with PCD had significantly lower nNO measurements than HC (p < 0.001), CF (p = 0.001) and asthma (p < 0.001). However, two participants with CSLD had very low levels (<6 nL/min) and there was no statistical difference in nNO values between this group and those with PCD (p = 0.11). One participant with CF and normal ciliary function and ciliary ultrastructure, had very low levels of nNO (<30 nL/min) measured using the NIOX® Flex and NIOX MINO®.

Receiver operating characteristic (ROC) curves were generated for the NIOX® Flex and NIOX MINO® at 2 ml/ sec and 5 ml/sec in cases with PCD versus participants without PCD (curves not shown) and cut-off values were determined for optimal sensitivity and specificity (Table 1). Using NIOX® Flex, a nNO cut off levels of 38 nL/min had 100% sensitivity and 95% specificity for distinguishing PCD patients from non-PCD patients. A cut off value of 30 nL/min when using the NIOX MINO® sampling at 5 ml/sec, provided 100% sensitivity and 95% specificity; using the same analyser, sampling at 2 ml/sec, a cut of value of 43 nL/min provided 100% sensitivity and 93% specificity for differentiating PCD patients from the other groups.

**Table 1:** Nasal nitric oxide cut-off values to discriminate between PCD and non-PCD groups using different analysers

	**NIOX® Flex Breath-hold**	**Niox Mino**_**5ml/sec**_	**Niox Mino**_**2ml/sec**_
		**Tidal breathing**	**Tidal breathing**
nNO cut-off value (nL/min)	38	30	43
Sensitivity%	100	100	100
Specificity%	95	95	93

### Comparison of nNO values between analysers

**Figure 1:**
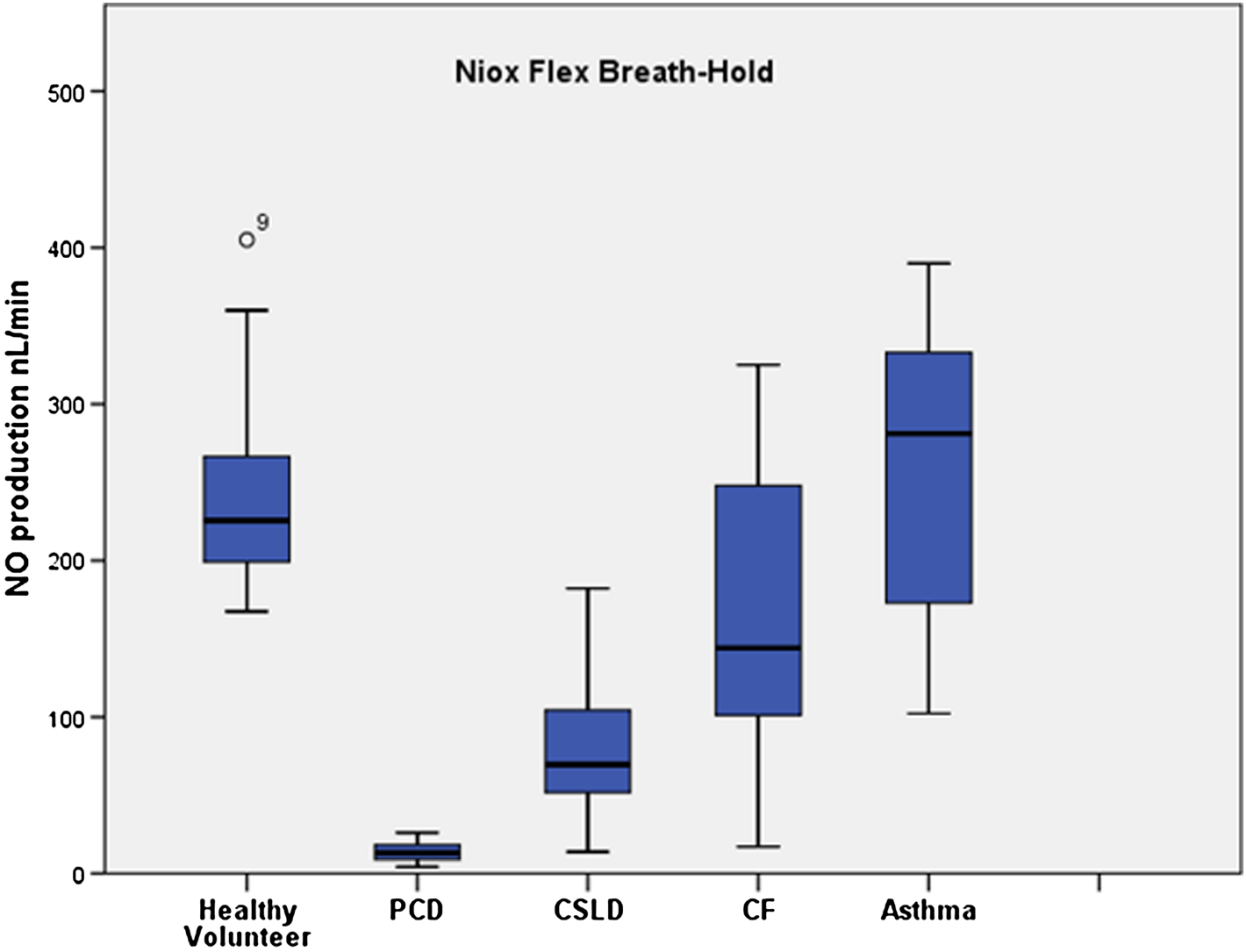
Box plots demonstrating nasal NO production (nL/min) measured by NIOX® Flex using a breath-hold manoeuver in healthy volunteers (n = 15), PCD (n = 11), CSLD (n = 6), CF (n = 6) and asthma (n = 9) (line: median, box: quartiles, whiskers: minimum and maximum.

**Figure 2:**
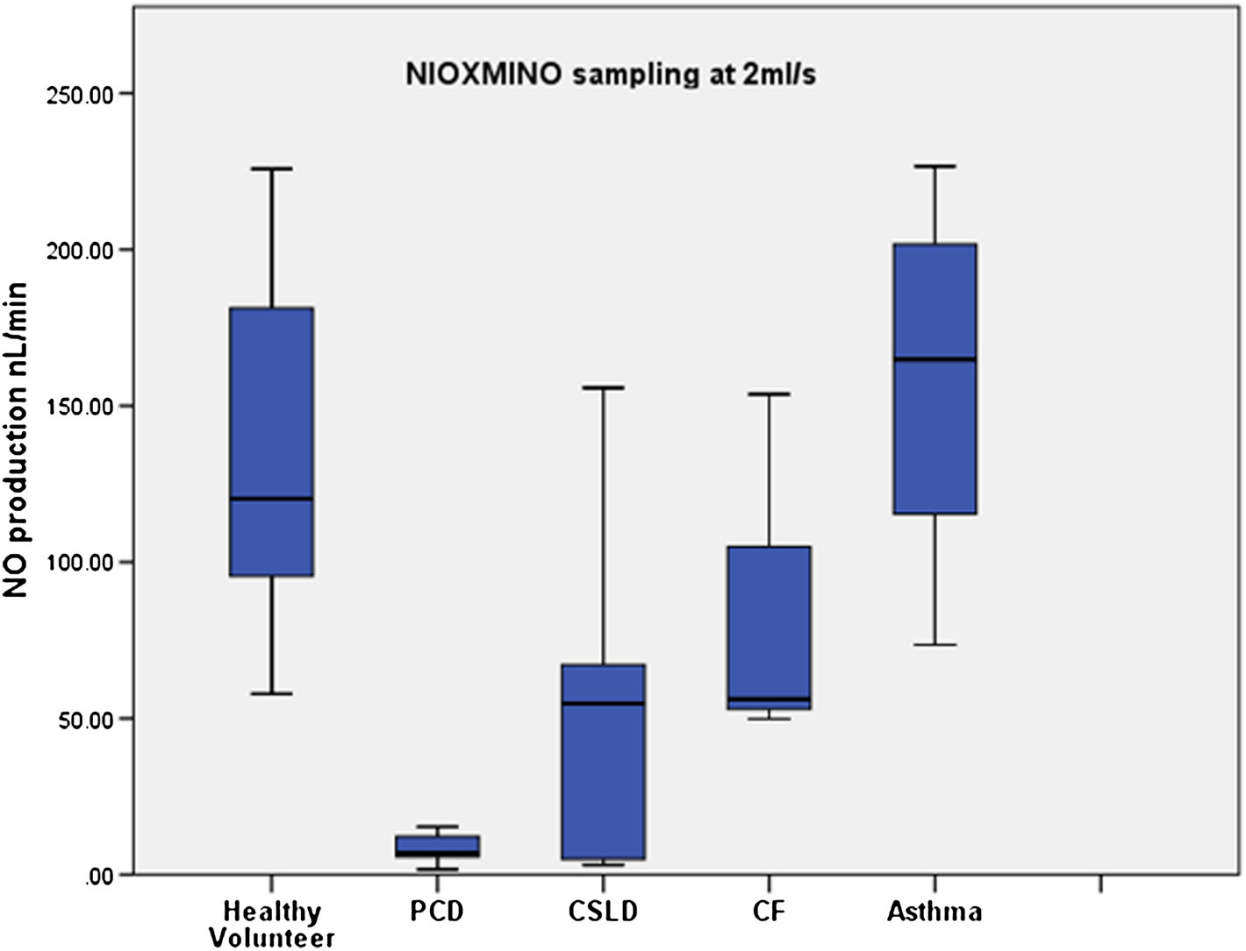
Box plots demonstrating nasal NO production (nL/min) measured by NIOX MINO® during mouth breathing with nasal sampling at 2 ml/ sec in healthy volunteers (n = 13), PCD (n = 9), CSLD (n = 5), CF (n = 3) and asthma (n = 8).

**Figure 3:**
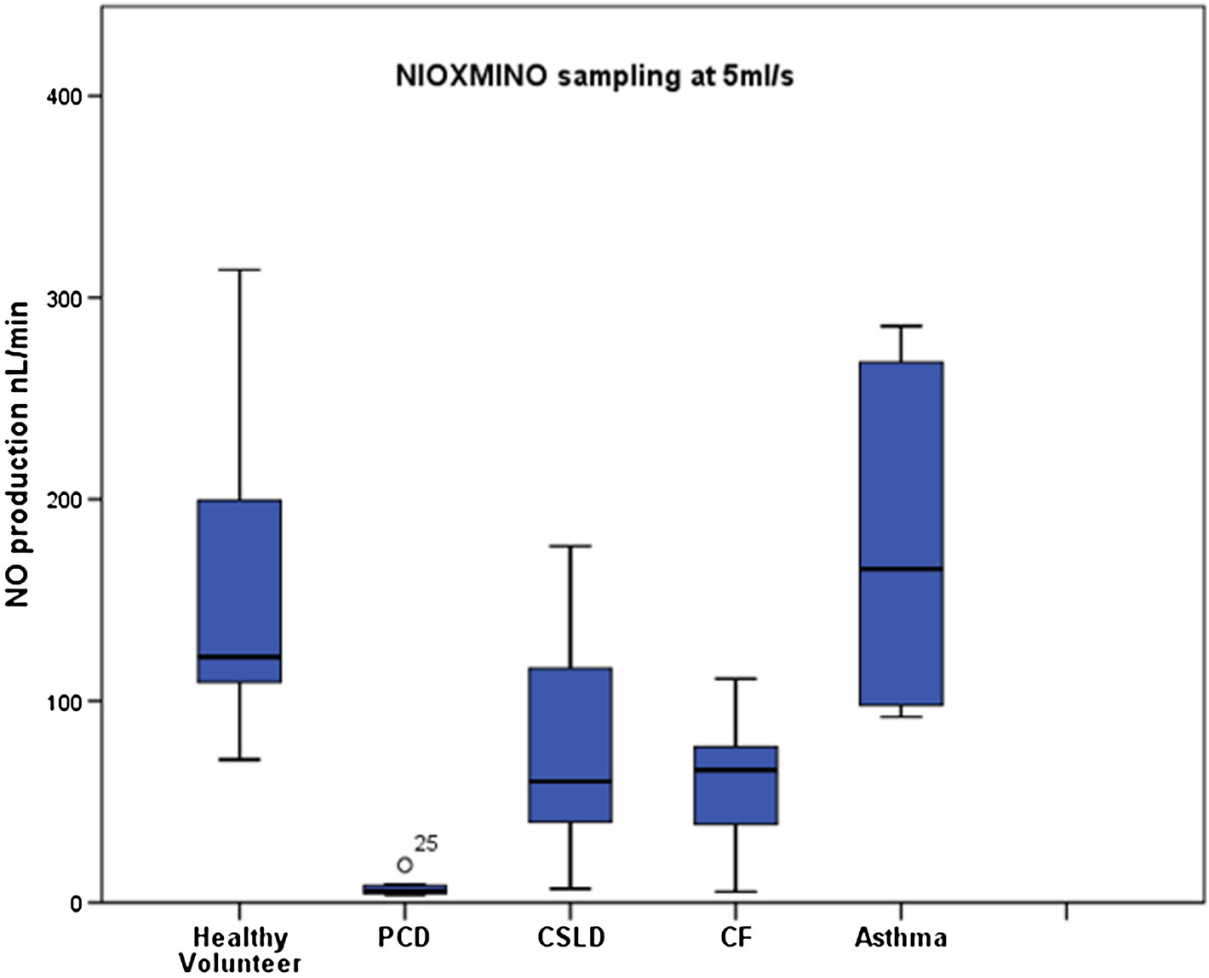
Box plots demonstrating nasal NO production (nL/min) measured by NIOX MINO® during mouth breathing with nasal sampling at 5 ml/ sec in healthy volunteers (n = 15), PCD (n = 12), CSLD (n = 7), CF (n = 5) and asthma (n = 9).

**Figure 4:**
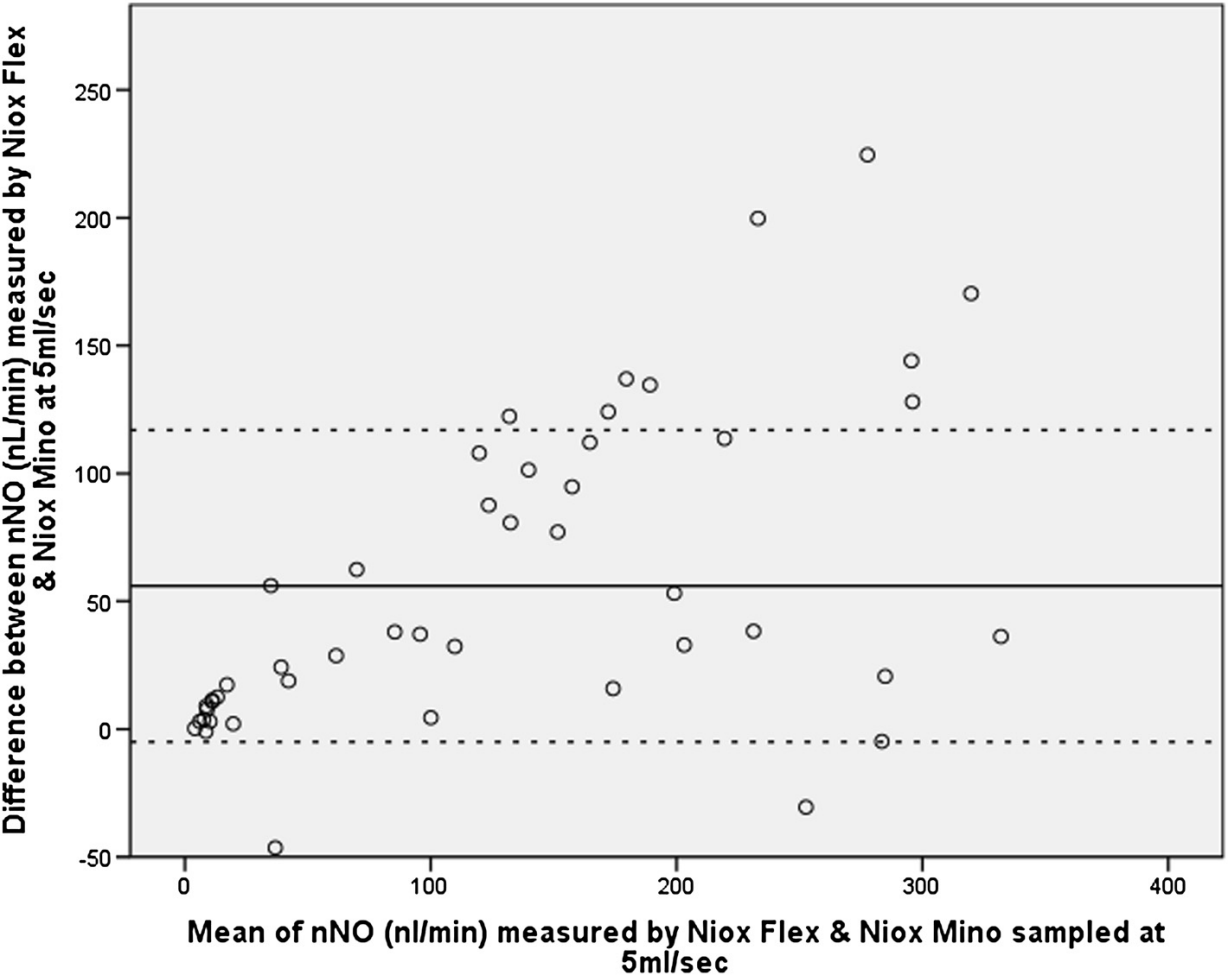
**Bland Altman plots comparing the NIOX® Flex with NIOX MINO® at 5 ml/sec.** Each point represents the difference between the nNO readings (nL/min) obtained from a patient using the two analysers versus the mean of the two measurements. The reference lines represent the mean (and 1.96*SD) inter-analyser differences.

Comparing analysers, within patients, the NIOX MINO® sampling at 5 ml/sec or 2 ml/sec consistently measured lower (p < 0.001, p < 0.001) than the NIOX® Flex (Figures 1, 2, 3). Pearson correlation analysis showed excellent linear association between nNO readings obtained by NIOX® Flex and NIOX MINO® at 5 ml/sec or 2 ml/sec (r = 0.93 p < 0.001 and r = 0.93 p < 0.001 respectively). The relationship between the nNO measured by NIOX® Flex and NIOX MINO® at 5 ml/sec were further investigated by Bland-Altman plot (Figure 4). The Bland-Altman plots show a high degree of difference between readings from the two analysers, particularly in participants with very high levels of nNO. However the patients with PCD all had low nNO levels, and this group all demonstrated low variability of readings between analysers.

### Reproducibility within-analyser

The intra-subject%CV showed good repeatability between three measurements taken during the same visit using NIOX® Flex (%CV = 9) and reasonable repeatability using the NIOX MINO® at 5 ml/sec (%CV = 15) and NIOX MINO® at 2 ml/sec (%CV = 14).

### Usability of the analysers

Prior to the study the nurse specialist and physiotherapist were highly experienced obtaining nNO readings from adults and children using the NIOX® Flex breath-hold protocol. They were unable to obtain readings with the NIOX MINO® using the breath-hold technique because even healthy adults were unable to breath-hold for the requisite time. Their assessment (Table 2) was that younger children were sometimes able to achieve nNO readings using the NIOX MINO® by using the mouth breathing technique with sampling at 5 ml/sec, even when acceptable reading were not obtained using the NIOX® Flex. However, the NIOX MINO® sampling at 2 ml/sec was a very lengthy procedure and fewer participants achieved it.

**Table 2:** Comparison of analysers and methods reflecting the opinions of the multidisciplinary specialist PCD team having conducted the research

	**NIOX® Flex 20 seconds**	**NIOX MINO®**
**Ease of use**	Easily used by children over 8 years and by some younger using breath hold (BH)	Breath hold difficult for 45 sec/ 90 sec by all ages. Tidal breathing (TB) easily achieved in 5 ml/sec sampling mode
**Time to complete 3 readings**	5-10 min (BH)	20-40 min (TB)
**Practical issues**	Needs stable environment and dedicated work space.	Needs to be used on a flat surface
	Poor portability	Excellent portability
	Breath-holding technique difficult for young children and advanced lung disease	Most people could manage the technique but it resulted in a dry mouth. Short sampling catheter
**Cost of machine**	Very Expensive. Approx £30,000	Expensive. Approx £2,100
**Need for calibration gases**	Yes. Approx £1,500 per annum	No- autocalibrates
**Need for maintenance service**	Yes. Approx £3,800 per annum	No
**Consumables**	Nasal sampling olives: £40 per 100	Nasal sampling olives: £40 per 100 patients
**Longevity**	Good	Sensor needs replacing after 100 or 300 readings (using our protocol 30 or 100 patients). Many readings failed but were still ‘counted’. Sensor expires after 1 year Approx. £1,215 for sensor 300.
**Range of nNO (ppb)**	25-2000 ppb	5-1700 ppb
**Effected by other electronic devices in room** e.g. mobile phones	No	Yes

The portability of the NIOX MINO® was considered a benefit by health care professionals involved in the study, although the need to be placed on a firm, flat surface and the short sampling tube were poorer ergonomic features.

## Discussion and conclusions

This study confirmed that the hand-held NIOX MINO® provides a reliable screening tool for PCD compared to the NIOX® Flex in this study population. This is consistent with two previous studies using a hand held device in PCD patients, one of which collected nasal gas via a tight fitting nasal mask during single expiration silent breathing or humming [15]. The other study compared a tidal breathing and breath-hold maneuvers (+/− velum closure) [18]. We were unable to obtain readings using the NIOX MINO® during breath-holding but found NIOX MINO® nNO readings taken during mouth breathing correlated well with measurements from the NIOX® Flex during breath-holding manoeuvres. This was particularly true for patients with PCD. Within our study population, we were more successful ( 96% versus 76% success) and quicker (15–30 minutes versus 20–40 minutes) measuring nNO using the NIOX MINO® at 5 ml/sec than at 2 ml/sec; we found no advantage for measuring at 2 ml/sec and would use 5 ml/sec in the future. A recent Danish study [18] using the NIOX MINO® similarly reported that the method recommended by ATS/ERS guidelines for measuring nNO [20] (aspiration of nasal gas during velum closure and breath-hold) was impossible at a sampling rate of 2 ml/sec, but they had a success rate of 70% during breath-hold at a rate of 5 ml/sec. This compared to 100% success when sampling at 5 ml/sec during tidal breathing [18].

We noted that all three methods gave reproducible results. Although the methods were well correlated, there were differences in the mean nNO levels between analysers. nNO production recorded by the NIOX MINO® was lower than that recorded by the NIOX® Flex. This is likely to reflect contamination of the nasal sample by lower airway gas during mouth breathing and short mucosal contact time. This demonstrates the need for different diagnostic cut-offs for different breathing methods. Using a stationary chemiluminescence NO analyser, a study [21] of 85 children including 20 with PCD similarly demonstrated that non-velum closure methods yielded lower values that during the velum closure technique recommended in ATS/ ERS guidelines [20]. As confirmed by our study, the authors reported open mouth tidal breathing provides reproducible and discriminatory nNO levels. Since measurement during tidal breathing is easier to perform, allowing its use in young children, standardisation of this approach and reference data is called for.

In our population, a cut-off value of 38 nL/min provided 100% sensitivity and 95% specificity using the NIOX® Flex during breath-hold. A cut-off of 30 nL/min using the NIOX MINO® (tidal breathing with sampling at 5 ml/sec) provided 100% sensitivity and 95% specificity. These cut off values are somewhat lower that those reported in a large study investigating the use of nNO as a screening test for PCD [9]. Leigh *et al.* determined a cut off value of 77 nL/min was 98% sensitive and >99.9% specific when screening patients at their PCD centre. The authors then used the same protocol to measure nNO in 155 patients referred for PCD diagnostic testing at six other hospitals. They found that this level correctly identified 70 of the 71 participants who were confirmed to have PCD. A study using the NIOX® Flex and NIOX MINO® to measure nNO during silent and humming exhalations [2,17] calculated lower cut-off values than in our study. This is likely to reflect greater contamination by lower airway gases during these exhalation manoeuvres. Importantly, the key findings of both manuscripts were in line with our paper, i.e. PCD patients have lower nNO than healthy controls or CF patients [9,17], and the hand-held device is effective for screening to differentiate PCD from other groups [17].

Whilst there is now substantial data to demonstrate that nNO measurement is helpful in guiding the diagnostic pathway, we need to recognize limitations of this measurement [10]. Standardized methods to measure nNO are not appropriate for younger children, precisely the age group that need targeting for diagnostic measurement. Sampling during tidal breathing provides a potential method in this group, but data is limited [22]. Low nNO has previously been described in patients with CF and other respiratory conditions. One of our patients with CF and negative PCD diagnostics had very low levels of nNO (NIOX® Flex 17 nL/min, NIOX MINO® 5 ml/Sec 5 nL/min). Two patients with CSLD also had low levels of nNO. Both had PCD excluded using protocols that follow ERS guidelines [2]. They had normal ciliary beat frequency and pattern before and following culture at air liquid interface. They also had normal ultrastructure by TEM. These data demonstrate that although nNO is useful as a screening test, it is not a substitute for formal assessment of ciliary function and assessment of ciliary ultrastructure. It is noteworthy that although all PCD patients in this study had very low levels of nNO, occasionally PCD patients with normal levels have been described [8,22,23]. This highlight that patients with a history strongly suggestive of PCD should not be excluded from further diagnostic evaluation on the basis of nNO.

An important observation by the authors has arisen from referrals to our diagnostic service where nNO has been measured by the referring team using NIOX MINO®. A number of patients have been noted to have extremely low levels of nNO at the referring hospital, but normal/ high levels when measured using the NIOX MINO® or NIOX® Flex at our centre. We suspect this might reflect leaks at the nares or excessive lower airway contamination, and highlights the need for training and standardised protocols if the NIOX MINO® is to be used as a screening test at satellite hospitals.

In summary, nNO measurements using the NIOX MINO® successfully discriminates PCD patients from healthy individuals and those with other lung conditions. Nasal NO values were reproducible and correlated well with the NIOX® Flex. However, we consistently recorded lower readings using the tidal breathing maneuver. The NIOX MINO® is easily portable and relatively cost-effective as compared to the desktop NIOX® Flex. Health care professionals and patients found it acceptable. Hand-held devices therefore provide a promising screening tool for PCD although further studies will be required to establish reference data for each breathing maneuver and analyser. One study has successfully attempted to standardise methodology across a number of collaborating sites with different analysers [9]. With the array of sampling techniques and analysers available, standardised protocols and cut off data should be developed. Due to the rarity of PCD it is likely that a coordinated international approach will be required to achieve this.

## Competing interests

The authors declare that they have no competing interests.

## Authors’ contributions

AH had the idea for the research and designed the study; EB, KG, RJ & JP undertook clinical testing and data recording; AH, HE and JSL undertook data analysis and interpretation; HE, WW & JSL recruited patients and are clinical leads for the PCD diagnostic service; all authors contributed to and approved the manuscript; JSL takes responsibility for the integrity of the study data and analysis. All authors read and approved the final manuscript.

## Pre-publication history

The pre-publication history for this paper can be accessed here:

http://www.biomedcentral.com/1471-2466/14/18/prepub
